# Ab Interno vs. Ab Externo Microcatheter-Assisted Circumferential Trabeculotomy in Treating Patients With Primary Open-Angle Glaucoma

**DOI:** 10.3389/fmed.2021.795172

**Published:** 2021-12-20

**Authors:** Weijia Zhang, Yiwei Wang, Chen Xin, Yang Sun, Kai Cao, Huaizhou Wang, Ningli Wang

**Affiliations:** ^1^Department of Ophthalmology, Beijing Tongren Eye Centre, Beijing Tongren Hospital, Capital Medical University, Beijing, China; ^2^Beijing Ophthalmology & Visual Sciences Key Laboratory, Beijing, China; ^3^Department of Ophthalmology, Henan Provincial People's Hospital, Zhengzhou, China; ^4^Beijing Institute of Ophthalmology, Beijing Tongren Eye Centre, Beijing Tongren Hospital, Capital Medical University, Beijing, China; ^5^Byers Eye at Stanford, Palo Alto, CA, United States

**Keywords:** primary open-angle glaucoma, intraocular pressure, minimally invasive glaucoma surgery, ab interno trabeculotomy, surgery

## Abstract

**Background:** Circumferential trabeculotomy have evolved from ab externo to ab interno approach. Both procedures may lower IOP, but it is unclear which maybe a superior approach.

**Purpose:** To compare the outcomes of ab interno and ab externo circumferential trabeculotomy in patients with primary open-angle glaucoma.

**Design:** Retrospective, comparative case series.

**Participants:** Primary open angle glaucoma patients undergoing ab interno (40 patients in Group 1) or ab externo (54 patients in Group 2) circumferential trabeculotomy, with about one half of them having prior incisional glaucoma surgery.

**Methods:** Outcomes including intraocular pressure (IOP), glaucoma medications and surgical complications were analyzed.

**Main Outcome Measures:** IOP, medications and surgical success defined as an IOP of ≤ 21 mmHg and a reduction of IOP ≥20% from baseline (criterion A) or IOP ≤ 18 mmHg and a reduction of IOP 20% from baseline (criterion B) with (qualified success) or without (complete success) medications.

**Results:** At 1 year, IOP decreased by 37.1% (26.0–14.8 mmHg) in Group 1 and 39.5% (28.5–15.1 mmHg) in Group 2. Medications decreased from 3.5 in Group 1 and 3.6 in Group 2 pre-operatively to 0.6 ± 1.0 and 0.3 ± 0.6 post-operatively, respectively. Success rates did not differ significantly between groups based on criterion A (complete and qualified success: 68.7 and 81.9% in Group 1, and 75.3 and 90.4% in Group 2, respectively) or criterion B (complete and qualified success: 58.2 and 79.3%in Group 1, and 69.5 and 88.4% in Group 2, respectively). For eyes with prior filtration surgeries, the mean percent reduction of IOP (41.7 ± 32.7% in Group 1, 39.7 ± 27.8% in Group 2, *P* = 0.724) and the mean medication reduction (2.9 ± 1.6 in Group 1, 3.4 ± 1.0 in Group 2, *P* = 0.454) were not significantly different.

**Conclusions:** Ab interno circumferential trabeculotomy achieved comparable outcomes to ab externo trabeculotomy and may be an effective surgical option for patients with primary open-angle glaucoma.

## Introduction

A primary risk factor for the development and progression of primary open-angle glaucoma (POAG) is elevated intraocular pressure (IOP), which is caused by an increased resistance to aqueous outflow. The juxtacanalicular tissue of the trabecular meshwork and inner wall of Schlemm's canal are thought to be the site of greatest resistance to aqueous outflow (1–3). Therefore, procedures that remove, disrupt, or bypass the trabecular meshwork and inner wall of Schlemm's canal can facilitate physiologic outflow and consequently lower IOP. Based on this knowledge, many canal-based procedures have been developed, including circumferential trabeculotomy (4–6).

Circumferential trabeculotomy procedures have evolved over years. Smith first described suture trabeculotomy over one third of the drainage angle in cadaveric eyes in 1960 (7). The technique was subsequently refined to achieve a 360°catheterization and trabeculotomy using suture material (4, 8) Another improvement was the introduction of a flexible illuminated microcatheter (iTrack, Ellex, Menlo Park, California, USA), which can verify the location of the illuminated cannula tip and facilitate cannulation by injecting viscoelastic material into Schlemm's canal to overcome the resistance caused by structural collapse or adhesions (5). Over the past two decades, ab externo circumferential trabeculotomy has become increasingly popular in the treatment of childhood glaucoma and open-angle glaucoma (5, 8–15). However, ab externo trabeculotomy requires the creation of a scleral flap, which has a few drawbacks. Recently, Grover et al. (6) introduced a conjunctival-sparing trabeculotomy through ab interno approach, gonioscopy-assisted transluminal trabeculotomy. In their preliminary study, success rates of the novel procedure ranged from 0.68 to 0.9 at 1 year in eyes with open-angle glaucoma (6). Subsequently, a couple of studies showed that ab-interno circumferential trabeculotomy alone or combined with cataract extraction provided a significant IOP-lowering effect in children glaucoma (16, 17) and open-angle glaucoma (18–24).

These two procedures may both reduce IOP, but little data is available to compare the two approaches. Previous studies showed comparable outcomes between ab externo and ab interno 360-degree trabeculotomy in primary congenital glaucoma eyes with clear cornea (17) and in patients with open-angle glaucoma (25). However, which option is safer and more effective remains unclear.

This study aims to compare the efficacy and safety of ab interno circumferential microcatheter-assisted trabeculotomy vs. ab externo circumferential trabeculotomy for POAG. Moreover, the two procedures will be particularly compared in treating patients with failed glaucoma surgery.

## Methods

### Study Design

A retrospective chart review was performed for POAG patients who underwent either microcatheter-assisted ab interno circumferential trabeculotomy between November 2017 and October 2018 (Group 1) or ab externo microcatheter-assisted trabeculotomy between March 2017 to February 2018 (Group 2) at Beijing Tongren Eye Center. The study was conformed to the tenets of the Declaration of Helsinki and approved by Institutional Ethics Committee of Beijing Tongren Hospital. Written informed consent was obtained from all subjects. All patients recruited in this study were diagnosed as POAG, and they had uncontrolled IOP, or presented progressive deterioration of visual field defects on maximum medication, or were intolerant to glaucoma medications. All enrolled subjects had comprehensive ophthalmic examinations pre-operatively. In those with prior filtration surgeries, the fistula site was examined carefully by gonioscopy to determine whether Schlemm's canal was intact or not. Anterior chamber angle and IOP were assessed during follow up visits, and anti-glaucoma medication usage was recorded at baseline and each follow up visit (1, 3, 6, and 12 months). Intraoperative and post-operative complications were also documented and analyzed. Combination glaucoma medications were evaluated according to the number of active agents in the medication. An IOP of ≥30 mmHg within 1 month of surgery was defined as an IOP spike (19).

### Surgical Procedure and Post-operative Care

All the surgeries were performed by a single surgeon (HZ Wang) using an illuminated ophthalmic microcatheter (iTrack, Ellex, Menlo Park, California, USA). The surgical procedure for ab externo microcatheter-assisted trabeculotomy was described previously (5, 11, 26). Briefly, following a fornix-based conjunctival peritomy, Schlemm's canal was exposed by a scleral cut down under a superficial scleral flap. In those with prior filtration surgeries, the scleral flap was made next to the earlier incision. Successful 360-degree catheterization was performed followed by circumferential trabeculotomy. In cases of incomplete catheterization owing either to obstruction or to misdirection, partial trabeculotomy was performed as follows. The conjunctiva was incised and a scleral cut down performed over the illuminated catheter tip, which was retrieved at the point of obstruction. Both exposed ends of the catheter were then grasped and pulled in a purse string fashion, thus breaking through the trabecular meshwork. In all cases, the scleral flap was sutured watertight. The anterior chamber was gently irrigated via a paracentesis when hyphema occurred.

The details of the ab interno procedure were also reported previously (6). A clear corneal incision was made at the temporal site. Viscoelastic material was injected into the anterior chamber. A paracentesis track, oriented tangentially, was then placed in the superonasal or inferonasal quadrant, serving as the entry site for the microcatheter. The microscope and the patient's head were then oriented to facilitate the best visualization of the nasal angle by the surgeon using a Swan-Jacob gonio lens. A 1- to 2-mm goniotomy was created in the nasal angle followed by circumferential catheterization and trabeculotomy. If the microcatheter stopped somewhere in Schlemm's canal, an ab interno cut down was performed to achieve a limited trabeculotomy, and the microcatheter was then placed in the opposite direction to excise the canal and to achieve as close to a 360-degree opening as possible. The viscoelastic substance was gently washed out, and the corneal wounds were hydrated to ensure a watertight closure.

Post-operatively, tobramycin-dexamethasone (TobraDex, Alcon, Rijksweg, Belgium) and pranoprofen (Pranopulin, Senju Pharmaceutical, Osaka, Japan) eye drops were used 4 times daily for 2–4 weeks. The topical steroids were tapered per the surgeon's discretion. Pilocarpine 2% (Bausch & Lomb, Rochester, NY, USA) was used 4 times daily for 3 months to prevent peripheral anterior synechia regardless of the IOP, and therefore was not considered as an anti-glaucoma medication during this time. IOP elevation within the first month after surgery has not been treated, unless IOP exceeded 25 mmHg. Prostaglandins and medications suppressing aqueous humor formation will be preferentially prescribed. Anterior chamber irrigation will be performed for cases with massive hyphema (blood filling one half of the anterior chamber) and IOP spike.

### Outcome Measures and Statistical Analysis

Surgical outcomes included IOP reduction and the reduction of glaucoma medications at the 1-, 3-, 6-, and 12-month follow-up visits as well as the rate of surgical success and occurrence of complications. Surgical success was defined as an IOP of ≤ 21 mmHg and a reduction of IOP ≥20% from baseline (criterion A) or IOP ≤ 18 mmHg and a reduction of IOP ≥20% from baseline (criterion B) with (qualified success) or without (complete success) glaucoma medications. Surgical failure was defined as IOP >21 mmHg or reduction of pressure <20% recorded on at least two occasions despite maximal medication, when further glaucoma surgery was required, or when visual acuity deteriorated to an absence of light perception.

Categorical variables (gender, side of eye, rate of eyes with prior filtration surgery, type of prior filtration surgery, proportion of pseudophakia, complications, and secondary surgical interventions) were tested by the chi-square test or Fisher exact test, as appropriate. The Mann-Whitney *U* test was performed for age, prior glaucoma surgery times, pre-operative IOP, pre-operative glaucoma medications and follow-up duration. Repeated measures variables were analyzed using mixed-effects models with Bonferroni-corrected significance level used for multiple comparison. The independent samples *t*-test was used to compare the absolute IOP reduction between groups, while the Mann-Whitney *U* test was used to compare the mean percent reduction of IOP and medication reduction between groups. The cumulative probability of success was analyzed using Kaplan-Meier survival analysis with the log rank test. A *P-*value of < 0.05 (2-tailed) was considered statistically significant.

## Results

### Demographics and Ocular Characteristics

One hundred and one patients with primary open-angle glaucoma were initially screened for inclusion in the study. Of these, 7 patients (2 with ab interno trabeculotomy, 5 with ab externo trabeculotomy) were lost to follow-up after the initial 1 week. Hence, data analysis was done for 94 eyes of 94 patients. The demographics and ocular characteristics are summarized in Table 1. Forty eyes of 40 patients who underwent ab interno circumferential trabeculotomy (Group 1) and fifty-four eyes of 54 patients who underwent ab externo circumferential trabeculotomy (Group 2) were included in this study. The two groups were comparable in terms of age (*P* = 0.513), the rate of right eyes (*P* = 0.680), proportion (*P* = 0.908) and times (*P* = 0.664) of prior filtration surgery, proportion of pseudophakic eyes (*P* = 0.426), pre-operative IOP (*P* = 0.204), number of pre-operative glaucoma medications (*P* = 0.897), extent of catheterization (*P* = 1.000) and follow-up duration (*P* = 0.512), but differed by sex (*P* = 0.049). The percentage of eyes with prior failed filtration surgery was 47.5% in Group 1 and 46.3% in Group 2. Fourteen patients with prior trabeculectomy, three with Ex-PRESS shunt implantation and two with Ahmed valve implantation were included in Group 1, and 22 patients with trabeculectomy and 3 with Ex-PRESS shunt implantation in Group 2. The extent of catheterization was not <270-degree, and 360-degree catheterization was performed in more than 90% of cases in both groups. The median follow-up duration was 12 months in both groups.

**Table 1:** Demographics of study population with baseline characteristics.

**Variables**	**Group 1**	**Group 2**	***P***
Eye/patients	40/40	54/54	
Age (year), mean ± SD	35.8 ± 12.3	34.2 ± 11.9	0.513^‡^
Female, *n* (%)	5 (12.5)	16 (29.6)	0.049^†^
Right eye, *n* (%)	18 (45.0)	22 (40.7)	0.680^†^
Rate of prior filtration surgery, *n* (%)	19 (47.5)	25 (46.3)	0.908^†^
Type of prior filtration surgery			
Trabeculectomy, *n* (%)	14 (35)	22 (40.7)	
Ex-press shunt, *n* (%)	3 (7.5)	3 (5.6)	
Ahmed valve, *n* (%)	2 (5.0)	0 (0)	
Prior filtration surgeries times, mean ± SD	0.6 ± 0.6	0.6 ± 0.8	
Pseudophakia, *n* (%)	1 (2.5)	0(0)	
Pre-operative IOP, (mm Hg) mean ± SD	26.0 ± 9.3	28.5 ± 10.7	0.204^‡^
Pre-operative medications, mean ± SD	3.5 ± 0.9	3.6 ± 0.7	0.897^‡^
Catheterization, *n* (%)
360°	37 (92.5)	49 (90.7)	
<360°	3 (7.5)	5 (9.3)	
330	0 (0)	3 (5.6)	
300°	1 (2.5)	2 (3.7)	
270°	2 (5.0)	0 (0)	
Follow-up duration in month, median (lower quartile, upper quartile)	12 (12, 12)	12 (12, 12)	0.512^‡^

*Group 1: Patients underwent abinterno circumferential trabeculotomy.*

*Group 2: Patients underwent abexterno circumferential trabeculotomy.*

† *Chi-square test.*

‡ *Mann-Whitney U test*.

### IOP and Medications

Table 2 demonstrates the results for IOP and glaucoma medications. Mean IOP decreased from 26.0 ± 9.3 mm Hg in Group 1 and 28.5 ± 10.7 mm Hg in Group 2 pre-operatively to 14.8 ± 3.5 mmHg at 12 months post-operatively in Group 1 and 15.1 ± 2.8 mmHg in Group 2. Both procedures significantly reduced IOP during follow-up (*P* < 0.001 for each post-operative visit compared to baseline). The mean percentage reduction of IOP in Group 1 was comparable to that in Group 2 (37.1% ± 26.5 vs. 39.5% ± 25.2%, *P* = 0.564).

**Table 2:** Results of IOP and medication.

	**Group 1**	**Group 2**
**IOP (mmHg)**		
Baseline	26.0 ± 9.3	28.5 ± 10.7
1 m	14.2 ± 3.9	14.5 ± 4.1
3 m	15.8 ± 4.0	13.8 ± 3.6
6 m	16.2 ± 4.0	14.5 ± 4.2
12 m	14.8 ± 3.5	15.1 ± 2.8
*P*-value	<0.001	<0.001
**Number of medications**		
Baseline	3.5 ± 0.9	3.6 ± 0.7
1 m	0.4 ± 1.0	0.3 ± 0.7
3 m	0.4 ± 0.9	0.2 ± 0.6
6 m	0.4 ± 0.9	0.3 ± 0.7
12 m	0.6 ± 1.0	0.3 ± 0.6
*P*-value	<0.001	<0.001

*Group 1: ab interno circumferential trabeculotomy group.*

*Group 2: ab externo circumferential trabeculotomy group.*

*Data are shown as means ± SD. Repeated measures variables were analyzed using mixed-effects models. Bonferroni-corrected significance level (0.01) was used for multiple comparison*.

Mean number of glaucoma medications decreased from 3.5 ± 0.9 in Group 1 and 3.6 ± 0.7 in Group 2 pre-operatively to 0.6 ± 1.0 at 12 months post-operatively in Group 1 and 0.3 ± 0.6 in Group 2. The mean number of glaucoma medications was significantly lower at all post-operative time points compared to baseline (*P* < 0.001) in both groups. Mean reduction in glaucoma medications was 3.0 ± 1.3 in Group 1 and 3.3 ± 1.0 in Group 2 after 12 months (*P* = 0.330).

### Success Rate

**Figure 1:**
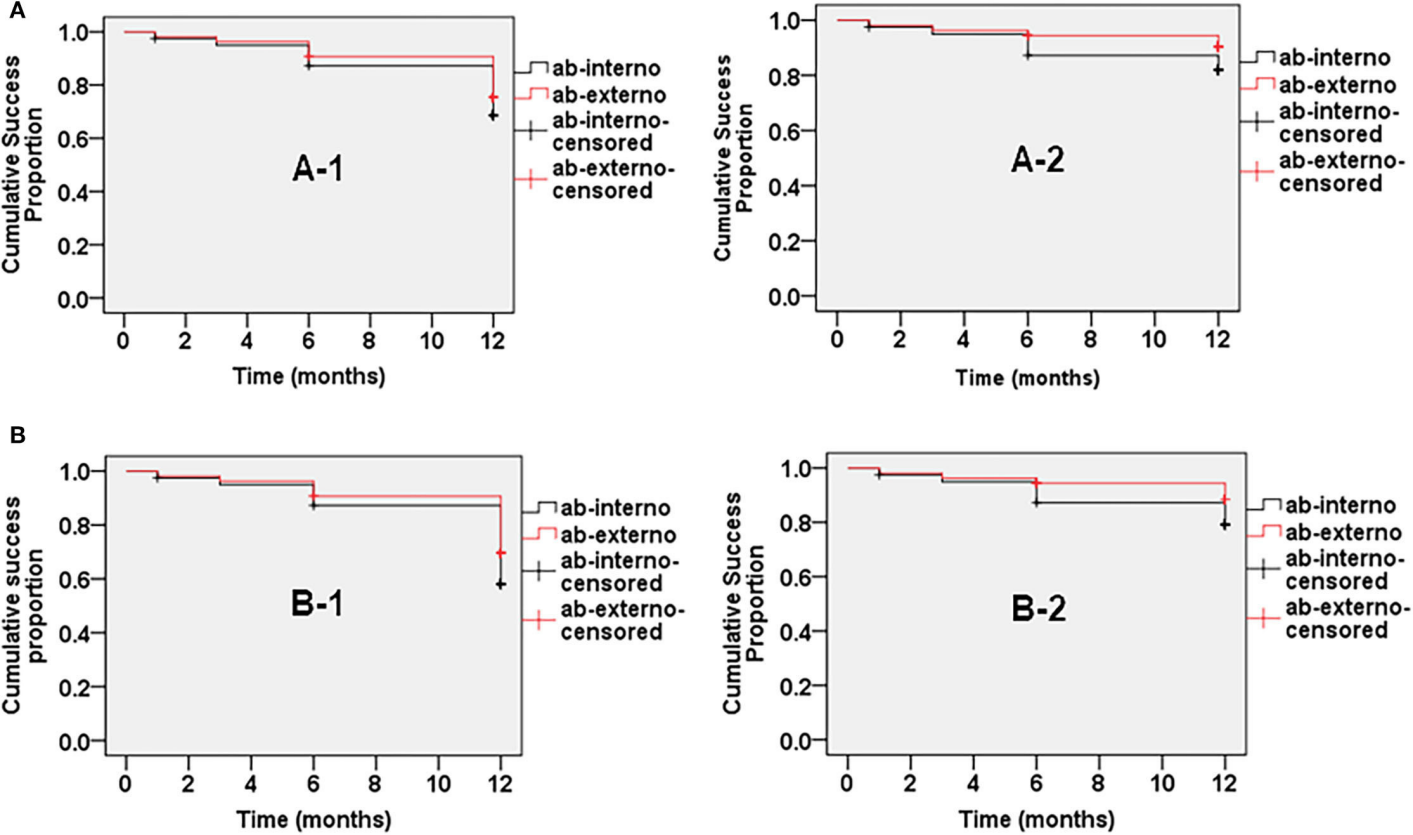
The cumulative success probabilities of abinterno (red) and ab externo (black) circumferential trabeculotomy. **(A)** Complete (A-1) and qualified (A-2) success (success criterion: IOP ≤ 21 mmHg and a reduction of IOP ≥20% from baseline). **(B)** Complete (B-1) and qualified (B-2) success (success criterion: IOP ≤ 18 mmHg and a reduction of IOP ≥ 20% from baseline).

Figure 1 shows the cumulative probabilities of success in Kaplan-Meier survival analyses. At 12-month follow-up, the cumulative probability of complete success was not significantly different between the two groups based on criterion A (68.7% in Group 1 and 75.3% in Group 2, *P* = 0.480) or criterion B (58.2% in Group 1 and 69.5% in Group 2, *P* = 0.272). Qualified success based on criterion A (81.9% in Group 1 and 90.4% in Group 2, *P* = 0.235) or criterion B (79.3% in Group 1 and 88.4% in Group 2, *P* = 0.229) also was not significantly different.

### Complications and Subsequent Interventions

The complications are summarized in Table 3. Patients in neither of the two groups developed vision-threatening complications, and complications did not differ significantly between the two groups. No complications occurred during surgery except for hyphema, which was also the most common post-operative complication in both groups. All patients demonstrated hyphema to different degrees on the first day after surgery. In most cases in both groups, hyphema resolved spontaneously by the second week. Twelve eyes (30.0%) in Group 1 and 19 eyes (35.2%) in Group 2 experienced IOP spike, which mainly occurred in the first post-operative week. Eyes with IOP spike, which was probably due to massive hyphema, were successfully treated by anterior chamber irrigation. Self-limited ciliochoroidal detachment appeared in 12 eyes (30.0%) in Group 1 and 15 eyes (27.8%) in Group 2. One eye in Group 2 developed circumferential wide ciliochoroidal detachment with anterior chamber shallowing, which lasted for more than 1 month and resulted in extensive anterior synechia.

**Table 3:** Post-operative complications and subsequent surgeries.

	**Group 1**	**Group 2**	***P***
**Post-operative complications**			
Hyphema	40 (100.0%)	54 (100.0%)	–
Spike of intraocular pressure	12 (30.0%)	19 (35.2%)	0.597^†^
In the first week after surgery	10 (25.0%)	13 (24.1%)	
In the second week after surgery	2 (5.0%)	6 (11.1%)	
Ciliary body detachment	12 (30.0%)	15 (27.8%)	0.814^†^
Shallow anterior chamber	0 (0)	1 (1.9%)	1.000^§^
**Subsequent surgeries**			
Trabeculectomy	3	1	
Ab-Internotrabeculotomy	0	2	
Goniosynechialysis	1	0	
Transscleralcyclophotocoagulation	1	1	
Total	5 (12.5%)	4 (7.4%)	0.635^†^

*Group 1: Patients underwent ab interno circumferential trabeculotomy.*

*Group 2: Patients underwent ab externo circumferential trabeculotomy.*

† *Chi-square test.*

§ *Fisher exact test*.

Five eyes in Group 1 and 4 eyes in Group 2 underwent additional glaucoma surgery to control IOP. In both groups, additional glaucoma surgery was generally performed within half a year post-operatively: 4 of these patients underwent filtration surgery, 3 patients with extensive adhesions in the trabecular meshwork incision underwent a second trabeculotomy or goniosynechialysis, and 2 underwent transscleral cyclophotocoagulation.

### Subgroup Analysis

**Figure 2:**
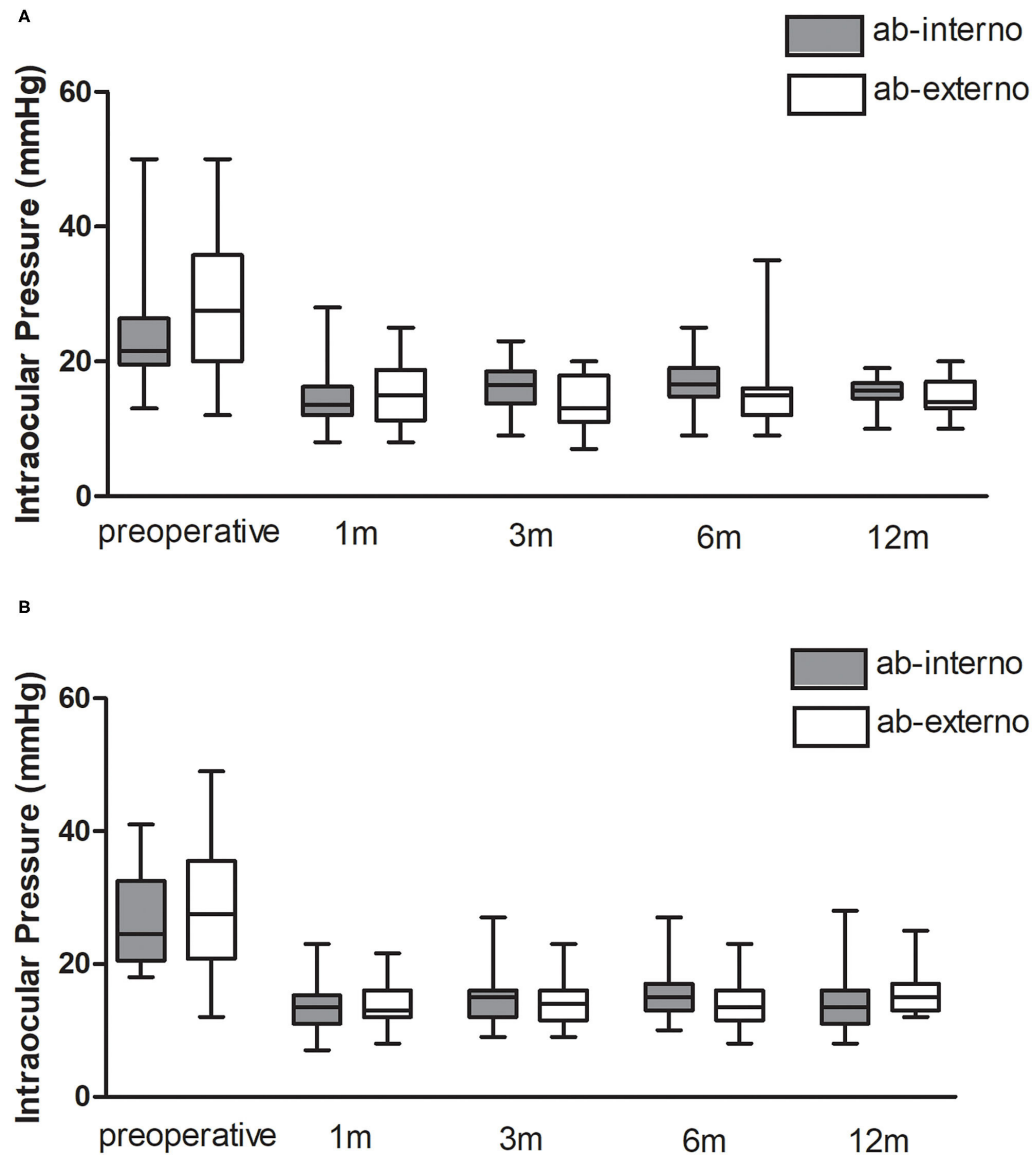
IOP comparison according to surgical approach. **(A)** patients without prior glaucoma surgery; **(B)** patients with prior glaucoma surgery.

Figure 2 is a chart that demonstrates the pre- and post-operative IOP among subgroups. For eyes without prior filtration surgeries, no significant difference between groups was found in the mean percent reduction of IOP (32.9 ± 19.2% in Group 1, 39.2 ± 23.1% in Group 2, *P* = 0.270) or mean medication reduction (3.0 ± 1.0 in Group 1, 3.2 ± 1.1 in Group 2, *P* = 0.686). For eyes with prior filtration surgeries, the mean percent reduction of IOP (41.7 ± 32.7% in Group 1, 39.7 ± 27.8% in Group 2, *P* = 0.724) and the mean medication reduction (2.9 ± 1.6 in Group 1, 3.4 ± 1.0 in Group 2, *P* = 0.454) were also not significantly different.

## Discussion

The current study compared the short-term outcomes of ab interno circumferential trabeculotomy to ab externo trabeculotomy in the treatment of POAG. Half of the study subjects had prior incisional glaucoma surgery. We found that both procedures significantly reduced IOP and glaucoma medication usage. Ab interno trabeculotomy is similar to ab externo trabeculotomy in in terms of safety and efficacy at 12 months in patients with POAG. For eyes with prior filtration surgeries, ab interno and ab externo procedure achieved similar IOP reduction.

Although ab externo circumferential trabeculotomy reliably reduces IOP, it does not spare the conjunctiva and may compromise future filtration surgery. Ab interno approaches have recently been drawning increasing attention. Grover et al. reported that gonioscopy-assisted transluminal trabeculotomy alone or combined with cataract extraction achieved 39.8% reduction in mean IOP in POAG and 56.8% reduction in secondary open-angle glaucoma at 12 months post-operatively (6). Another study of patients with POAG found a 37% mean percentage decrease of IOP at 24 months and a decrease of 1.43 glaucoma medications (23). Moreover, gonioscopy-assisted transluminal trabeculotomy was reported to be safe and successful in treating 60–70% of open-angle patients with prior incisional glaucoma surgery (20). The results in this study were consistent with those in previous studies.

Yalinbas et al. (25) compared the results from 33 eyes with ab externo 360-degree suture trabeculotomy and 23 eyes with ab interno 360-degree suture trabeculotomy and showed similar outcomes. The sample size in our study was relative larger. Unlike their study, about half of the participants had prior glaucoma surgery in our study, and we used illuminated microcatheter rather than suture. Similarly, the overall percent reduction of both IOP and medication numbers in Group 1 was comparable to that in Group 2. Unexpectedly, we noticed that mean IOP at 3 and 6 months after ab interno trabeculotomy was higher than after ab externo trabeculotomy. The cause of the difference was unclear. El Sayed et al. (27) reported a filtering bleb in some cases after ab externo trabeculotomy. We can also occasionally find localized mild subconjunctival elevation overlying the scleral flap in Group 2, which disappeared within 3 months. We speculated that there might be a potential sub-conjunctival drainage pathway during the early stage after ab externo trabeculotomy, which may contribute to additional IOP-lowering effect.

Johnson et al. (28) reported that the region of the Schlemm's canal was filled with extracellular material due to underperfusion of the trabecular meshwork, and that the size of the canal decreased after successful filtration surgery. Although nearly half of the patients had undergone prior filtration surgery in our study, we found that no extra effort was required for circumferential cannulation. Grover et al. (20) reported similar results. Moreover, ab interno trabeculotomy as well as ab externo trabeculotomy was effective for eyes with prior surgery in this study. In these cases, the distal outflow system quite possibly remained intact or amenable to rejuvenation. However, accurate assessment of the distal drainage pathway remains a challenging clinical issue.

In terms of complications, both ab interno and ab externo circumferential trabeculotomy were safe for POAG patients. Hyphema resolved spontaneously by the second week in most cases. About one third of the study subjects experienced an IOP spike in the first 2 weeks. The exact causes of post-operative IOP spike are still unclear. In previous studies, post-operative use of corticosteroid was supposed to be the cause of IOP spike (6, 29). While the patients in our study did receive corticosteroid post-operatively, this would not explain why IOP spike usually occurred within the first few days after surgery. Shi et al. reported that IOP spike was related to the closure of cyclodialysis clefts. We found similar phenomenon in our experience. Moreover, intraoperative hyphema and poor reserve in the post-trabecular outflow pathway were related to IOP spike. When the post-trabecular outflow system cannot compensate for the influence to outflow drainage caused by bleeding, IOP spike may occur. A couple of studies reported that IOP spike was related to surgical failure after circumferential trabeculotomy (21, 29, 30). We suggest that close monitoring on IOP spike is needed. The frequency of ciliochoroidal detachment was lower than that reported by Sato et al. (31). The discrepancy may result from the difference in the frequency of anterior segment optic coherence tomography examination.

Our study has several limitations. First, this study follows a retrospective design; thus prospective randomized controlled trials are needed to further clarify this issue. Second, we did not perform multiple analysis of risk factors because of the small sample size and the complexity of the patients. Third, this study only addresses short-term results. Future studies need longer follow-up to evaluate both procedures.

To conclude, ab interno trabeculotomy is similar to ab externo trabeculotomy in terms of the safety and efficacy profiles for POAG, even for cases with prior failed filtering surgery. Ab interno circumferential trabeculectomy showed promise as a reasonable surgical choice for patients with primary open-angle glaucoma.

## Data Availability Statement

The original contributions presented in the study are included in the article/supplementary material, further inquiries can be directed to the corresponding author/s.

## Ethics Statement

The studies involving human participants were reviewed and approved by Institutional Ethics Committee of Beijing Tongren Hospital. The patients/participants provided their written informed consent to participate in this study.

## Author Contributions

NW and HW: conception, design, and interpretation. WZ, YW, HW, and CX: data collection. YW, WZ, and KC: data analysis. YW, WZ, HW, and YS: writing paper. All authors contributed to the article and approved the submitted version.

## Conflict of Interest

The authors declare that the research was conducted in the absence of any commercial or financial relationships that could be construed as a potential conflict of interest.

## Publisher's Note

All claims expressed in this article are solely those of the authors and do not necessarily represent those of their affiliated organizations, or those of the publisher, the editors and the reviewers. Any product that may be evaluated in this article, or claim that may be made by its manufacturer, is not guaranteed or endorsed by the publisher.
